# Recent Synthetic Studies Leading to Structural Revisions of Marine Natural Products

**DOI:** 10.3390/md7030314

**Published:** 2009-07-13

**Authors:** Yoshihide Usami

**Affiliations:** Osaka University of Pharmaceutical Sciences, 4-20-1 Nasahara, Takatsuki, Osaka 569-1094, Japan; E-Mail:usami@egly.oups.ac.jp; Tel.: +81-72-690-1083; Fax: +81-72-690-1005

**Keywords:** structural revision, stereoselective, synthesis, marine natural product

## Abstract

Because of the highly unique structures of marine natural products, there are many examples of structures that were originally proposed based on spectral analyses but later proven incorrect. In many cases, the total syntheses of the originally proposed structures of marine natural products has confirmed their incorrectness and the subsequent total syntheses of the newly proposed structures proved the revised structures. This review will show such cases appearing after 2005 and demonstrate how the true structures were elucidated.

## 1. Introduction

In the last several decades, research has expanded from land to ocean in order to find new leads for drug candidates. Because the ocean occupies almost 70% of Earth’s surface, it offers an unlimited possibility of biological and chemical diversities [1–3]. Given such a background, marine natural products chemistry has been progressing at an unprecedented rate, resulting in a multitude of discoveries of carbon skeletons and molecules hitherto unseen on land.

However, the sad truth is that structures originally proposed based on detailed modern spectral analyses often include misassignments. Because marine natural products often possess unusual structures, such as a large membered ring or a spiro-fused ring, they are prone to such misassignments and only synthetic studies could provide the correct structures. However, the total syntheses of marine natural products require extremely high level work in organic chemistry. The original purposes of the total synthesis are to elucidate the absolute configuration, to develop effective synthetic routes that would supply materials for biological assays, or to take up the challenge of constructing complicated molecules. Nevertheless, synthetic organic chemists sometimes end up with incorrect structures of target molecules after multi-step stereoselective total syntheses. They must re-examine the original data of the compounds in order to deduce consistent structures and restart the synthesis to yield the true structures of natural product molecules. We had experienced an exciting series of synthetic studies on the antitumor compound pericosine A [4–8], which will be mentioned briefly later.

**Figure 1:**
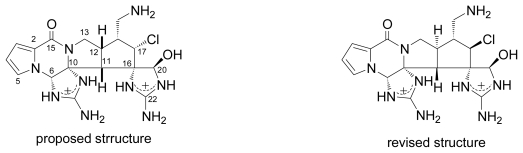
Proposed and revised structures of palau’amine.

Although there are many excellent reviews [9–13] of the synthesis of marine natural products, in 2005, an excellent and encouraging review on misassignments and structural revisions was published by Nicolaou and Snyder [14]. It included a database of example cases as well as their own narrative of revisions of bioactive marine natural products diazonamide A and azaspiracid-1. Herein we will review recent synthetic studies of marine natural products that led to structural revisions after 2005 except for palau’amine shown in Figure 1 [15], which has been described in another review [16].

## 2. Examples of Syntheses Leading to Structural Revisions of Marine Natural Products

### 2.1. Macrocyclic Marine Natural Products

Macrocyclic structure is one of the typical features of many marine natural products. Because of the large membered rings, macrocyclic marine natural products are highly flexible and this flexibility has made conformational analysis a difficult task.

**Scheme 1:**
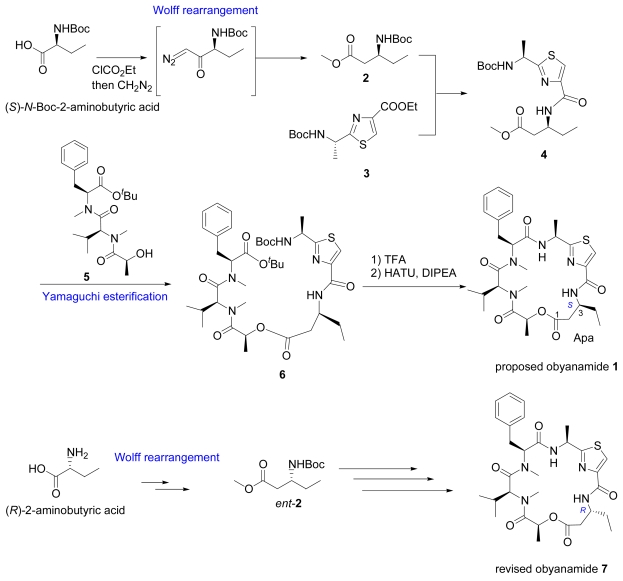
Synthesis of proposed and revised structures of obyanamide.

A cytotoxic depsipeptide designated as obyanamide (**1**) was isolated from the marine cyanobacterium *Lyngbya confervoides*, as shown in Scheme 1. Compared to related natural products, in the originally proposed structure **1** it had an unexpected (*S*)-configuration at C-3. The stereochemistry at C-3 in the Apa (aminopentanoic acid) part was deduced by chemical degradation and comparison of the hydrolyzated mixture with synthetic standards due to the limited amount of available material [17]. The total synthesis of **1** reported by Li and co-workers in 2005 is summarized in Scheme 1. Methyl (*S*)-*N*-Boc-3-aminopenatanoate (**2**), derived from (*S*)-2-aminobutyric acid via a Wolff rearrangement, was combined with ester **3** to afford amide **4**. A Yamaguchi esterification between **4** and **5** afforded ester **6**, which was cyclized to give the desired **1**. But this total synthesis suggested the need for structural revision [18]. Since all other related compounds isolated from the *Lyngbya* species had *R*-configuration in the β-amino acid residues, the same research group thought the revised structure should be **7**. Thus they carried out the total synthesis of **7** using the same strategy with methyl (*R*)-*N*-Boc-3-aminopenatanoate (*ent-***2**) derived from (*R*)-2-aminobutyric acid via a Wolff rearrangement, and proved that **7** is indeed the correct structure [19].

**Scheme 2:**
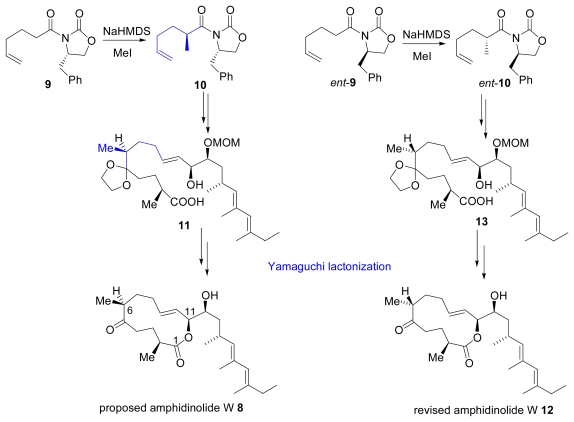
Synthesis of proposed and revised amphidinolide W.

The isolation of amphidinolide W (**8**), a new cytotoxic 12-membered macrolide from the dinoflagellate *Amphidinium* sp., was reported in 2002. The absolute configuration at C-6 was determined by the advanced Mosher’s method, utilizing the secondary alcohol that was obtained by degradation of the Baeyer-Villiger oxidation product of amphidinolide W [20]. Ghosh and co-workers synthesized proposed structure **8** via a Yamaguchi macrolactonization, as illustrated in Scheme 2. Chiral oxazolidone **9** was alkylated to **10** stereoselectively. Compound **10** was transformed into **8** via intermediate **11**, showing that the structure of **8** did not match the reported data.

After a careful review of the ^1^H-NMR spectra of natural amphidinolide W and the synthesized compound **8**, they found significant discrepancies between the chemical shifts in the C2-C11 ring region. Since they had synthesized **8** [(2*S*,6*S*)-isomer] and another epimer at C-2 [(2*R*,6*S*)-isomer] and neither of them corresponded to amphidinolide W, they hypothesized that the stereoisomer at C-6 **12** would be the revised structure. Then **12** was synthesized via intermediate **13** with *ent***-9** as a starting material. This total synhesis proved that **12** was the correct structure of amphidinolide W. In that study, they also synthesized another epimer at C-6, which is (2*R*,6*R*)-isomer [21,22].

**Scheme 3:**
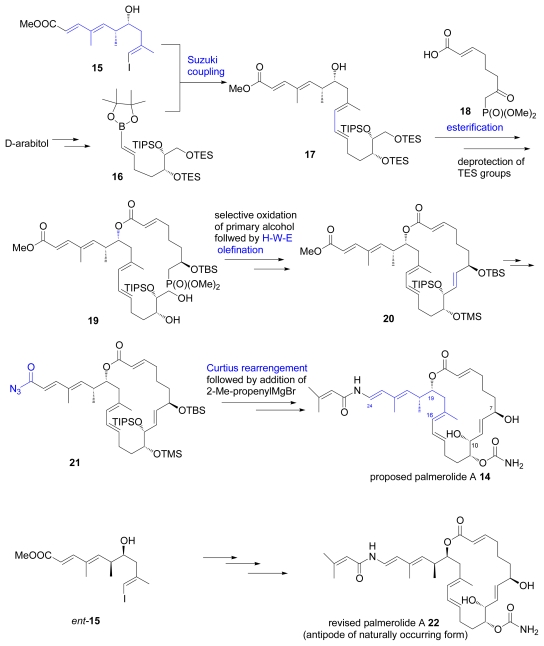
Synthesis of proposed and revised structures of palmerolide A.

Palmerolide A is a macrolide isolated from the Atlantic tunicate *Synoicum adareanum.* It exhibits selective cytotoxicity to UACC-62 (melanoma), HCC-2998 (colon cancer), and RXF 393 (renal cancer) cell lines [23]. The relative stereochemistry of the originally proposed structure **14** was assigned on the basis of a combined analysis of coupling constants and the NOESY spectrum. The absolute configurations at C7 and C10 were assigned by the advanced Mosher’s method, as in the previous example. De Brabander and co-workers achieved the total synthesis of **14** via the Horner-Wadsworth-Emmons olefination of C8 and C9 as a key step for macrocyclization as summarized in Scheme 3. Chiral vinyliodide **15** was coupled with borate **16**, which was derived from _D_-arabitol, to afford alcohol **17**. Then acid **18** was esterified with **17** to **19.** After selective oxidation of primary hydroxyl group in **19**, the resulted aldehyde was cyclized to **20** via H-W-E olefination. Methyl ester **20** was transformed to azide **21**, which was then converted into **14** via Curtius rearrangement followed by addition of 2-methylpropenylmagnesium bromide. As the data for **14** did not match that reported for the natural product, it was concluded that a structural revision was required.

After careful review of the NMR analysis of natural palmerolide A, De Brabander and co-workers thought that the relative stereochemistry C-10-C11 and C-19-C20 seemed reliable, but believed that the stereochemistry from C11 to C19 was doubtful. Then they set on next target **22** and carried out the total synthesis starting from *ent-***15**. Since data of synthesized **22** was identical with those of palmerolode A except for the CD-spectrum, the correct structure of palmerolide A were determined and also the absolute configuration were elucidated [24]. A few months later, Nicolaou *et al.* reported the synthesis of **14** and naturally occurring enantiomer *ent*-**22** via ring-closing metathesis, constructing the C8-C9 double bond as the key reaction [25,26].

**Figure 2:**
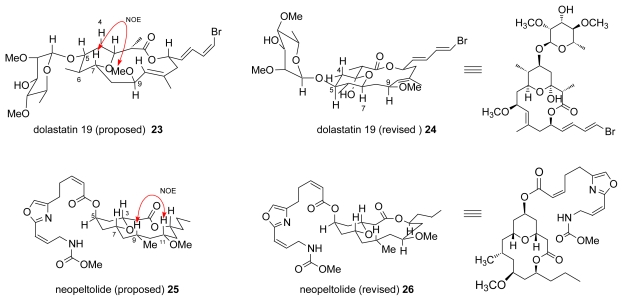
Structures of 14-membered lactone marine natural products.

The following two cases of dolastatin 19 and neopeltolide are quite similar. Both of them have a 14-membered lactone fused to a pyran ring with the C3-C7 skeleton, as illustrated in Figure 2.

**Scheme 4:**
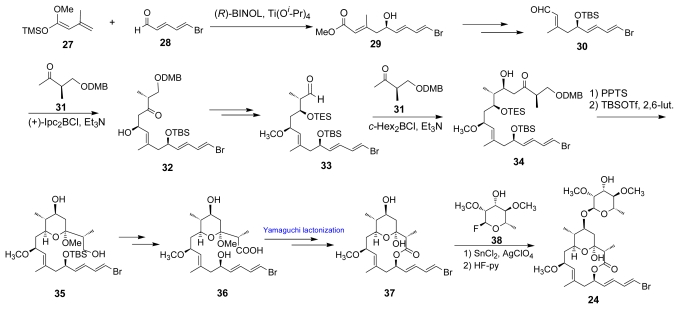
Synthesis of revised dolastatin **19**.

Dolastatin (**19**) is a cancer cell growth inhibitor isolated from the sea hare *Dolabella auricularia* collected in the Gulf of California [27]. The initial stereostructure **23** was determined by careful spectral analysis. It must be noted that the presence of a NOESY cross peaks between H7/9-OMe in dolastatin (**19**) as illustrated in Figure 2 seems to contradict the revised structure **24**. Paterson *et al.* proposed the revised structure **24** that has a different configuration on C5, C6, C7, and C13, through detailed conformational analysis of ^1^H-NMR spectra and calculation of the lowest energy of the proposed molecule **23**, and also through common bacterial biogenesis of related polyketides. The stereoselective total synthesis of **24** was achieved in 23 steps in 1.7% total yield as summarized in Scheme 4. An asymmetric Ti(O*^i^*Pr)_4_-(*R*)-BIBOL catalyzed aldol condensation between **27** and **28** yielded **29**. Aldehyde **30**, that was derived from **29**, was applied to a 1,4-*syn* boron mediated aldol reaction with ketone **31** to give **32**. After transformation of **32** to aldehyde **33**, **33** was applied to another 1,4-*syn* boron mediated aldol reaction with ketone **31** to give β-hydroxyketone **34**. Compound **34** was treated with PPTS with trimethylorthoformate in MeOH to give **35** with a tetrahydropyran-ring formation and a methyl ether function. After transformation into carboxylic acid **36**, 14-membered lactone **37** was formed via a Yamaguchi lactonization. This total synthesis was completed by coupling of **37** with fluoroalchohol **38** to afford desired **24**, thereby elucidating the relative and absolute configurations [28].

**Scheme 5:**
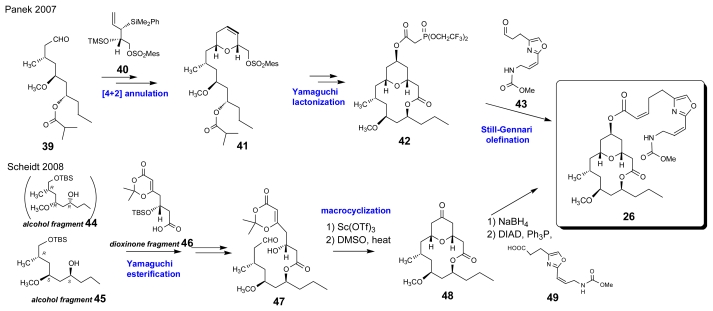
Synthesis of neopeltolide.

Neopeltolide derived from *Neopeltidae* sponge is a potent inhibitor of human cancer cell lines, such as A-549 and NCI-ADR-RES, and the P388 murine leukemia cell line [29]. Toward the end of 2007, Panek’s group and Scheidt’s groups reported independently the synthesis of neopeltolide, as shown in Scheme 5. Their accounts are quite similar.

After initial efforts to synthesize the proposed structure **25**, Panek’s group set **26** as their next target based on close inspection of available spectral data and its structural homology to leucascandrolide A. The dihydropyran **41**, which was formed by triflic acid promoted [4+2] annulations of aldehyde **39** with allylsilane **40**, was applied to a Yamaguchi macrolactonization. Stereoselective oxymercuration of the double bond in the dihydropyran moiety of the Yamaguchi lactonization product followed by acylation with bis(2,2,2-trifluoroethyl)phosphoacetic acid afforded phosphonoacetate **42**. A Still-Gennari olefination between **42** and aldehyde **43** yielded **26**. This total synthesis gave the correct relative and absolute configuration of neopeltolide [30].

**Scheme 6:**
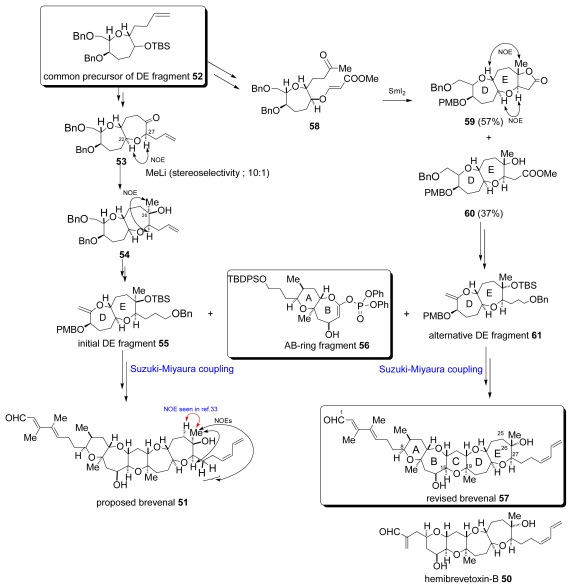
Total synthesis of brevenal.

A few months after the report by Panek’s group, Scheidt and co-workers reported the synthesis and structural revision of neopeltolide. Like in Penek’s study, they synthesized **25** along the same route summarized in Scheme 6, starting from a coupling between alcohol fragment **44** and dioxinone fragment **46** using a Yamaguchi esterification followed by the scandium(III) triflate catalyzed macrolactonization. Recognizing that **25** was not neopeltolide, they postulated the correct structure of **26** and started the synthesis from a Yamaguchi esterification between alcohol fragment **45** and **46**. Macrolactonization of **47** using Sc(OTf)_3_ as a catalyst gave lactone **48**, which was finally converted to **26** via stereoselective NaBH_4_ reduction followed by a Mitsunobu reaction with carboxylic acid **49** [31]. The authors mentioned the difficulty of assigning the relative configuration of such a flexible macrocyclic natural product, whose originally proposed stereostructure around C9-C11-C13 in **25** was assigned from the NOESY cross peak between H9 and H11, as shown in Figure 2 [29].

### 2.2. Etheric Marine Natural Products

Polyetheric or etheric compounds are well-known marine natural products. The isolation of brevenal from cultures of the dinoflagellate *Karenia brevis*, which is structurally related to hemibrevetoxin-B (**50**) but is almost half the size of the most well known metabolites of the same microorganisms called brevetoxins, was reported [32,33]. It is noteworthy that brevenal is not toxic to fish and antagonizes the toxic effects of brevetoxins in fish.

Fuwa and co-workers attempted to perform the total synthesis of proposed structure **51** with their strategy of building a pentacyclic core via the advanced Suzuki-Miyaura coupling between AB and DE ring fragments as illustrated in Scheme 6. The common precursor of the DE fragment **52** was transformed into ketone **53**, whose relative configuration was confirmed by the NOESY cross peak between H22/H27. Ketone **53** was stereoselectively methylated with methyllithium affording a diasteromeric mixture of alcohols in a 10:1 ratio. The NOESY cross peak between H27 and the methyl group at C26 suggested the stereochemistry of the major product **54**, which was transformed into the initial DE fragment **55**. Alkylborane prepared from **55** *in situ* coupled with the AB ring enol phosphate **56** in the presence of Cs_2_CO_3_ and Pd(Ph)_3_. The coupled product gave desired **51** through multistep transformations; however, it was not identical with natural brevenal [34]. Then, the authors reviewed the NMR spectra of **51** and the natural product and found a significant deviation of the chemical shift values around C26 tertiary alcohol. In addition, there was no description in the literature [33] of NOEs between 26Me/H27 and 26Me/H28α,β, which were observed in the NOESY spectrum of **51**. For the related marine natural product hemibrevetoxin-B (**50**), the configuration at C18, which corresponded to C26 in brevenal, was assumed by biosynthetic considerations [35].

Then, Fuwa *et al.* postulated that natural brevenal (**57**) should be the epimer at C26 of **51** and restarted the synthesis of brevenal. The common precursor of the DE fragment **52** was transformed into ketone **58**, and this was subjected to reductive cyclization with SmI_2_ to form a 7-membered ether ring with the desired stereochemistry in a mixture of lactone **59** (57%) and hydroxylether **60** (37%). The relative configuration of **59** was confirmed by NOESY analysis where cross peaks H22/H27 and 26Me/H23 were observed. Both products **59** and **60** could be converted into the alternative DE fragment **61**, which reacted with AB-ring enol phosphate **56** to afford the cross-coupled product. This finally yielded revised brevenal (**57**) via similar multiple sequences (Scheme 6) [36].

**Scheme 7:**
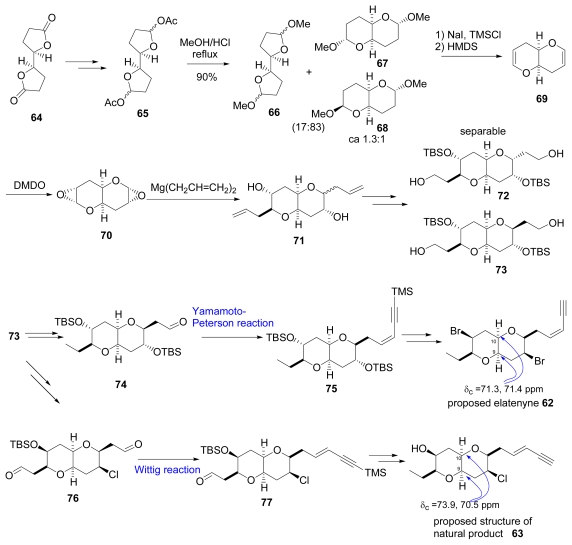
Synthesis of proposed structures of a metabolite from *Laurencia* sp.

Elatenyne was isolated from the red alga *Laurencia elata* in 1986 and the proposed structure **62** is shown in Scheme 7 [37]. Isolation of structurely related natural product **63** from *Laurencia majuscula* was reported in 1993. The proposed structure was determined by NMR analysis and by comparison of spectral data with elatenyne [38]. Recently Burton and co-workers attempted to perform the total synthesis of elatenyne **62** and **63** as summarized in Scheme 7. The known bislactone **64** was transformed in to a mixture of anomeric acetate **65**. Treatment of **65** with acidic methanol under reflux gave a mixture of **66**, **67** and **68**. Then the mixture was transformed solely into **69**. Compound **69** was oxidized stereoselectively with dimethydioxirane to bis(epoxide) **70**, that was then reacted with diallylmagnesium to give a diastereomeric mixture of bis(allylated)diols **71**. The resulted inseparable mixture **71** was converted to separable alcohols **72** and **73**. Purely isolated **73** led to desired **62** via Yamatoto-Peterson reaction from **74** to **75**.

**Figure 3:**
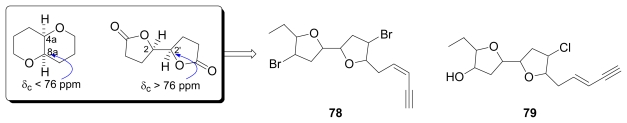
Proposed and reproposed structures of elatenyne.

In the same report **63** was synthesized from **73** via **77**, which was prepared from aldehyde **76** by a Wittig reaction. But the data of **62** and **63** did not agree with those of natural products, suggesting both of them required structural revision [39,40]. The authors then reviewed the spectral data of reported natural products and synthesized **62** and **63** carefully, paying special attention to the ^13^C-NMR chemical shift values at the ring juncture particularly pointed out in Scheme 7 and Figure 3. During the synthetic study of **62** and **63**, they synthesized a large number of pyrano[3,2-*b*]pyrans and 2,2′-bisfuranyl compounds and found that ^13^C-NMR chemical shift values at the ring juncture fall into two distinct groups. When the δ value was larger than 76 ppm, the compound belongs to a 2,2′-bisfuranyl-group, otherwise it belongs to a pyrano[3,2-*b*]pyran-group. The C-9 or C-10 chemical sifts values of synthesized **62** and **63** were δ 71.3, 71.4 ppm (for **62**) and δ 73.9, 70.5 ppm (for **63**). These evidences supported the structures of synthesized compounds **62** and **63**. But natural elatenyne and the eneyne from *L. majuscula* had larger ^13^C-NMR chemical shift values. In the original literatures ^13^C-NMR chemical shift values for C-9 and C-10 of **62** and **63** were reported as δ 80.0 and 79.5 ppm for **62** and δ 79.2 and 77.9 ppm for **63** [37,38]. Therefore Burton and co-workers proposed the structures of elatenyne and the eneyne from *L. majuscula*as to be **78** and **79** respectively shown in Figure 3.

### 2.3. Other Examples

**Scheme 8:**
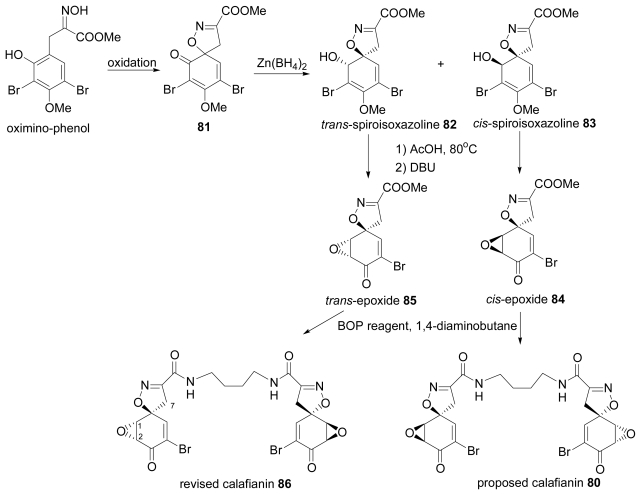
Synthesis of proposed and revised calafianin.

Calafianin (**80**) (proposed structure), a spiroisoxazoline marine natural product, was isolated from the Mexican sponge *Aplysina gerardogreeni*. The relative configuration around the spiroisoxazoline moiety was deduced from NOE experiments, observing 2% enhancement of H7 signal when H1 was irradiated and 5% enhancement of H7 signal when H2 was irradiated [41]. The total synthesis of racemic **80** was conducted by Nishiyama’s group, as summarized in Scheme 8. *Trans*- and cis-piroisoxazolines **82**, **83** was constructed as the key intermediate by oxidation of oximino-phenol affording **81** followed by reduction with Zn(BH_4_)_2_. Compound **80** was prepared from **83** via *cis*-epoxide **84** but the spectral data were not identical with those of the natural product. Applying the same reaction sequence to the *trans*-epoxide **85**, which was derived from **82**, furnished revised calafianin **86**. It must be noted that synthesized **86**, which had identical spectral data to natural calafianin, exhibited NOE correlation between H1 and H7 [42,43]. After the revision of the relative configuration of calafianin by Nishiyama’s group, the asymmetric synthesis for the assignment of (+)-calafianin was reported by Bardhan *et al.* [44].

**Scheme 9:**
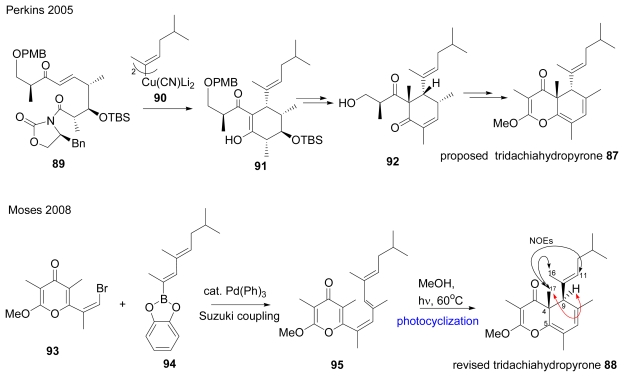
Synthesis of proposed and revised tridachiahydropyrone.

Tridachiahydropyrone, a structurally interesting fused bicyclic γ-dihydropyrone-containing natural product, was isolated from the Caribbean sacoglossan mollusc *Tridachia crispata.* Its stereochemistry was assigned as **87** as shown in Scheme 9 by NMR analysis. NOE experiments in particular suggested the *cis-*configuration between H9 and the 17-methyl group [45]. The total syntheses of proposed structure **87** and revised structure **88** were carried out by independent research groups, as summarized in Scheme 9. In order to determine the absolute configuration Perkins and co-workers synthesized **87**. Compound **89** was reacted with cuprate **90** with cyclization affording cyclohexenol intermediate **91** that was then stereoselectively methylated.

The stereochemistry of the subsequent intermediate enone **92** was established by X-ray crystallography. The asymmetric total synthesis of **87** completed via a four-step process from **92** confirmed that **87** was the incorrect structure [46]. In 2008, Moses and co-workers reported the racemic total synthesis of revised **88.** The revised structure was arisen from their biosynthetic hypothesis of photochemical, conrotatory 6π elctrocycloaddition of hypothetical precursor **95**. Suzuki coupling between vinylbromide **93** and borate **94** gave **95**, which was applied to subsequent photochemical electrocyclization yielding desired **88**. The authors noted not only the same NOE correlation between H9 and the 17-methyl group in **88** as described in the literature [45] but also a more intense NOE between 17-methyl/16-methyl and 17-methyl/H11 [47].

**Scheme 10:**
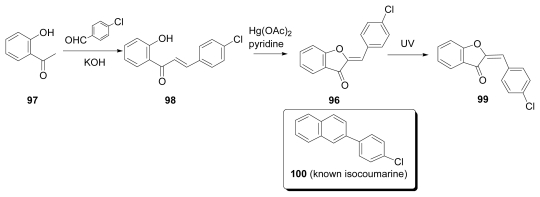
Synthesis of proposed structures of 4′-chloroaurone.

Another example of the synthesis of 4′-chloroaurone **96**, a bioactive metabolite from the marine brown alga *Spatoglossum variabile* [48], was achieved by Subbaraju as shown in Scheme 10. 2-Hydroxyacetophenone (**97**) was reacted with 4-chlorobenzaldehyde under the basic condition to give chlorochalcone **98**, which was then treated with mercury (II) acetate to afford the desired **96**.

Since the spectral data of **96** did not match with those of reported 4′-chloroaurone, *Z*-**96** was then photoisomerized to thermodynamically more stable *E*-isomer **99**. But **99** did not agree with 4′-chloroaurone either. After careful analysis of the spectral data for natural 4′-chloroaurone, the authors found that the data agreed well with the reported data of known 3-(4′-chlorophenyl)-isocoumarin (**100**) [49].

### 2.4. Our Experiences with Pericosines

**Scheme 11:**
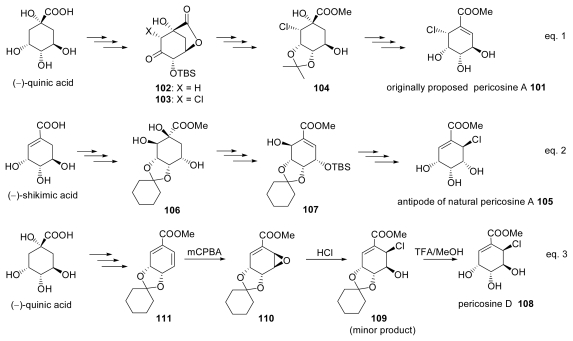
Synthesis of pericosines.

The isolation of pericosines A and B as cytotoxic metabolites of the fungus *Periconia byssoides* OUPS-N133 originally separated from the sea hare *Aplysia kurodai* was reported in 1997 [50]. As the absolute configuration of pericosine A was not determined, we performed the total synthesis of the originally proposed structure of pericosine A **101** as shown in Scheme 11 (Equation 1). Known lactone **102** derived from (−)-quinic acid was chlorinated to chloroketone **103** in a stereoselective manner. Conversion of **103** to **101** was achieved via intermediate **104** and we found that **101** was incorrect structure of pericosine A [4,5]. In 2006, we reported the first total synthesis of the antipode of revised pericosine A (−)-**105** and established its absolute configuration [6]. Revised structure **105** was presented after detailed analysis of NMR data for the natural product and several synthesized compounds related to pericosines. Total synthesis of **105** starting from (−)-shikimic acid involved a stereoselective dihydroxylation with catalytic osmiumtetroxide and trimethyamine-*N*-oxide yielding **106** and a stereoselective induction of chlor atom against alcohol **107** as shown in Scheme 11 (Equation 2). Since the synthesized **105** showed identical spectral data to natural pericosine A except for the sign of specific rotation, it was proved to be antipode of the natural product. The following year, we synthesized the natural form (+)-**105** [7].

In 2007, a full account of the isolation and structure elucidation of pericosines A–E was reported by the original researchers [51]. In that paper, the structure of pericosine A was revised based on our synthesis and new compound pericosine D **108** was reported. The following year, we synthesized **26** from chlorohydrine **109** that was obtained as a minor product of the ring opening reaction with hydrogen chloride of epoxide **110** derived from (−)-quinic acid via unstable diene **111**, and elucidated the absolute configuration of natural **108** [52] by a synthetic approach, as shown in Scheme 11 (Equation 3). We also revised the spectral data of the natural product because synthesized **108** possessing the proposed structure showed different spectral data from the natural product [51]. We concluded that the originally reported pericosine D must have a different relative configuration from the reported acetonide, which had relative stereochemistry corresponding to **108**. After completing the synthesis of (−)-pericosine B [53] and the improved synthesis of (+)-pericosine A **105** and C [54], we will undertake the new challenge of elucidating the correct structure of originally reported pericosine D.

## 3. Summary

In our review of the literature that has appeared in the last couple of years, we can witness numerous examples of misassignments of structures of marine natural products. Clearly, we still have much to learn about marine natural products possessing unusual structural features. We saw what synthetic chemists had to go through to overcome such formidable situations. Each and every total synthesis is extremely high level work. We recognized again the importance of synthetic studies for structure elucidation, as well as the need to supply marine-derived materials or to chemically modify molecules to increase pharmacological activity, in the development of marine-derived drugs.
